# Estimating age in elderly individuals: a morphological and quantitative approach using the pubic symphysis

**DOI:** 10.1007/s00414-026-03751-y

**Published:** 2026-04-10

**Authors:** Cindy Mansour, Nicholas Márquez-Grant, Eugénia Cunha, Javier Lescure, María García Velasco, María Benito Sánchez

**Affiliations:** 1 Laboratory of Forensic Anthropology, Department of Legal Medicine, Psychiatry and Pathology, Faculty of Medicine, Universidad Complutense De Madrid, Madrid, Spain; 2 Cranfield Forensic Institute, Cranfield University, Cranfield, MK43 0AL UK; 3 Centre for Functional Ecology, Laboratory of Forensic Anthropology, Department of Life Sciences, University of Coimbra, Calçada Martim de Freitas, Coimbra, 3000-456 Portugal; 4 Physical Anthropology Unit, Department of Biodiversity, Ecology and Evolution, Faculty of Biology, Complutense University of Madrid, Madrid, Spain; 5 Instituto Nacional de Medicina Legal e Ciências Forenses, IP, Lisbon, Portugal

**Keywords:** Forensic science, Forensic anthropology, Age estimation, Adult individuals, Spanish sample, Portuguese sample, Pubic symphysis

## Abstract

Estimating age in elderly adults is particularly challenging in forensic anthropology due to accelerated skeletal degeneration that varies greatly among individuals and the reduced precision of existing methods for advanced age groups. This study examines eight pubic symphyseal features in 228 documented individuals aged 60 or older from contemporary skeletal collections in Portugal and Spain, and incorporates pubic bone weight to evaluate its relationship with increasing age. Regression tree analysis identified two late-adult phases (Phases 1 and 2), which align with refined versions of the Suchey–Brooks, Hartnett, and Berg Phases VI and VII. The method demonstrated strong intra- and interobserver reliability (ρ = 0.77–0.97 and ρ = 0.58–0.75), minimal bias (− 4.75 to + 3.12), and acceptable inaccuracy (1.78–8.12 years). Pelvic bone weight showed a moderate negative correlation with age (ρ = −0.44, *p* < 0.01), and no significant bilateral differences or effects from hip prostheses were observed. This refined method provides a reproducible approach for estimating age in elderly individuals, improving overall accuracy and applicability. Further validation with additional samples from other populations is recommended.

## Introduction

Throughout life, bone undergoes continuous remodeling, which is a coordinated process of bone resorption, reversal, and formation within basic multicellular units. Osteoclasts remove old bone, and osteoblasts subsequently deposit new lamellar bone. The resulting secondary osteons and cement lines reflect the cumulative history of remodeling and provide essential indicators for assessing age-at-death, trauma healing, and other skeletal characteristics relevant to forensic interpretation [1]. Age estimation of human remains, a component of the biological profile, relies largely on morphological changes produced by this process, which progresses at different rates across the skeleton. These processes are strongly modulated by genetics, and by factors such as nutrition, hormonal changes, reproductive history, occupational stress, habitual physical activity, chronic disease, medication use, substance abuse, and socioeconomic conditions, which differ markedly within and between individuals and populations [2–5]. Therefore, chronological age is not consistently reflected in skeletal morphology; forensic anthropologists can only estimate biological age instead [6]. Methods for estimating age in older adults include assessing the pubic symphysis, auricular surface, acetabulum, cranium, sacrum, and sternal rib ends. They evaluate the cumulative effects of remodeling, ossification, and surface degeneration that emerge with advancing age [2, 7–14]. Additionally, these methods do not provide sufficiently accurate age estimates for older adults. In many cases, the only information available in the literature is that an individual was “older than 65 years.” Considering life expectancy trends in regions such as Europe and the United States, individuals who reach 65 years of age may live for several additional decades, often 30 years or more [15]. This reality poses a significant challenge, not only because age is a fundamental component of the biological profile in the analysis of unidentified remains, but also because a notable proportion of cases submitted for forensic anthropological assessment involve older individuals. Therefore, improving the accuracy of age-estimation methods for the elderly should be regarded as a priority.

Among several anatomical regions used to estimate adult age, the pubic symphysis remains one of the most widely studied and applied structures due to its predictable, though variable, sequence of morphological changes. Multiple methods have been developed for the pubic symphysis [3, 10, 16, 17], with the Suchey–Brooks method [8] remaining the most widely used in both forensic and bioarchaeological contexts [18, 19]. It is considered most reliable for the first three phases; however, beyond these early phases, its accuracy decreases substantially, particularly among those aged over 45–50 years old [16, 20]. Degenerative skeletal changes such as irregular marginal lipping, increased porosity, trabecular bone reduction, and heterogeneous ossification patterns become more pronounced and less predictable. Recognizing these limitations, Hartnett [16] and Berg [17] revised the Suchey–Brooks model and refined phase descriptions to address morphological variation observed in older adults using more contemporary skeletal collections from North America and Southeastern Europe (the Balkans).

Given that the pubic symphysis has long been used for age estimation, the question arises: which additional pubic traits can be further explored to improve the precision of phase-based systems and achieve narrower age ranges? To address this question, the present study investigates additional pubic-based age features using contemporary and identified samples from the Portuguese and Spanish osteological collections and measures the weight of the pubic symphysis to verify its correlation with increased age. Although Portugal and Spain curate well-documented osteological collections, few studies have focused specifically on age estimation in elderly individuals [21–27].

On this basis, the present study evaluates the effectiveness of the documented pubic-based traits (Table 2) for estimating age in older adults, and investigates their relevance and potential value in forensic anthropology. Three objectives guide this work:

1. **To develop a standardized quantitative scoring system** describing advanced morphological traits observed in the pubic symphysis.
2. **To incorporate pelvic bone weight** as an additional quantitative variable and to verify its correlation with age and morphological degeneration.
3. **To refine the upper-age phases of existing systems**, specifically replacing Suchey–Brooks Phase 6 with Phase 1 and Hartnett Phase 7 with Phase 2, through narrower diagnostic criteria, improved age ranges, and bone-weight thresholds.

## Materials and methods

### Sample

The study sample comprised 228 identified adult individuals (120 females; 108 males), aged 60 to 100 years, derived from two contemporary skeletal collections from Portugal and Spain. Individuals were selected based on the availability of well-preserved pelvic bones, documented biological sex, and known age-at-death. Both collections represent modern cemetery samples.

A. Portuguese Sample (UC Collection).

The Portuguese subset included 154 individuals: 77 females (mean age ± SD = 84.12 ± 9.31 years) and 77 males (mean age ± SD = 78.71 ± 9.40 years), all from the 21 st Century Identified Skeletal Collection curated by the Laboratory of Forensic Anthropology at the University of Coimbra. This collection contains more than 300 fully documented adult skeletons, born between 1896 and 1937, and deceased between 1982 and 2012, and is widely used in forensic anthropological research [28].

B. Spanish Sample (UCM Collection).

The Spanish subset consisted of 74 individuals: 43 females (mean age ± SD = 77.44 ± 8.28 years) and 31 males (mean age ± SD = 75.77 ± 9.27 years) from the Contemporary Identified Skeletal Collection housed at the Laboratory of Forensic Anthropology, Universidad Complutense de Madrid. This collection contains 238 fully documented skeletons (of which 107 are adults, born between 1881 and 1923 and died between 1978 and 1983) and also serves as an important research reference [29].

For this study, individuals aged 60 and older were selected to enhance and refine a method for elderly individuals. Aging in the skeleton is difficult to translate into a precise chronological age as it is inherently variable. Not assuming that more morphological degeneration or biological threshold occurs exactly at 60 but the decision to focus specifically on individuals over 60 years of age is because multiple studies have shown that after approximately the sixth decade or in advanced adulthood, there is a marked increase in skeletal degenerative processes (degenerative joint disease, cortical bone thinning, trabecular deterioration, vertebral compression, and dental senescence) and variability [30, 31]. Individuals with well-preserved left and right pubic symphyses were analyzed when available; if one side was missing or damaged, the contralateral side was used. Additionally, pelves with evidence of surgical intervention, such as prosthetic implants at the femoral head level, were included to determine whether they influenced degenerative scores or bone weight. However, those with traumatic and clear pathological conditions were excluded. Table 1 summarizes the age and sex distribution for both collections. Each entry represents a single individual, for whom one or both pelvic bones were examined, depending on preservation.

**Table 1:** Age-at-death and biological sex distribution of the study sample for each skeletal collection (*N* = 228)

	The 21 st Century Portuguese Identified Skeletal Collection housed at UC			The Contemporary Spanish Identified Skeletal Collection housed at UCM			Individual Total
Age Interval	Male *N* (%)	Female *N* (%)	Total *N* (%)	Male *N*(%)	Female *N*(%)	Total *N* (%)	
60–70	14	6	20	6	10	16	36
71–80	26	13	39	12	14	26	65
81–90	28	33	61	10	16	26	87
91–100	9	25	34	3	3	6	40
Total	77	77	154	31	43	74	**228**

UCM, University Complutense of Madrid collection; UC, University of Coimbra collection

### Methodology

#### Development of the scoring system and pelvic bone weight

Method development was conducted in two stages. First, the Portuguese sample (UC) was used to design and calibrate a scoring system tailored to morphological changes in elderly individuals which was then applied to the Spanish sample (UCM) to evaluate reproducibility.

**Fig. 1:**
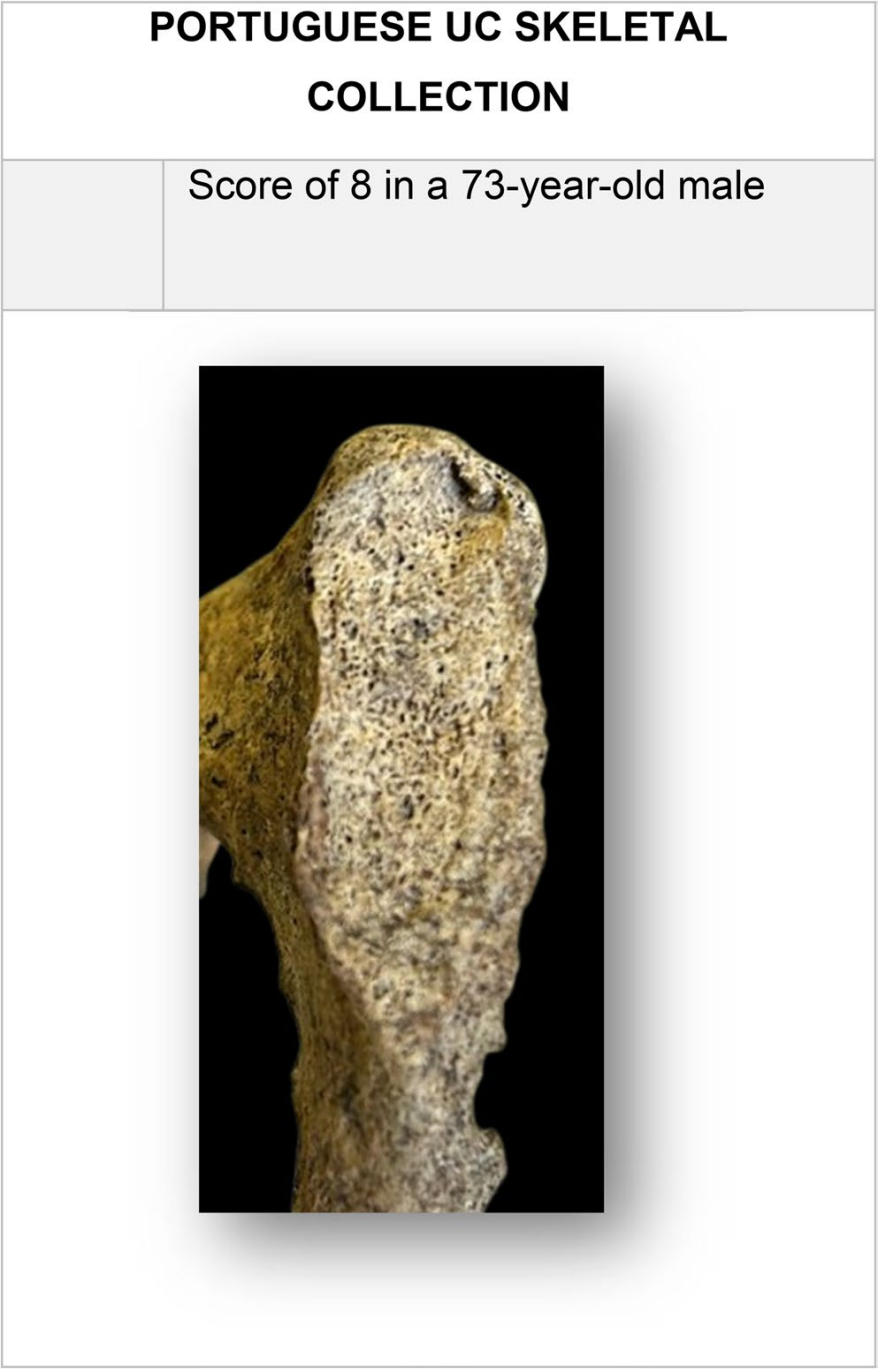
Score of 8 in a 73-year-old male

**Fig. 2:**
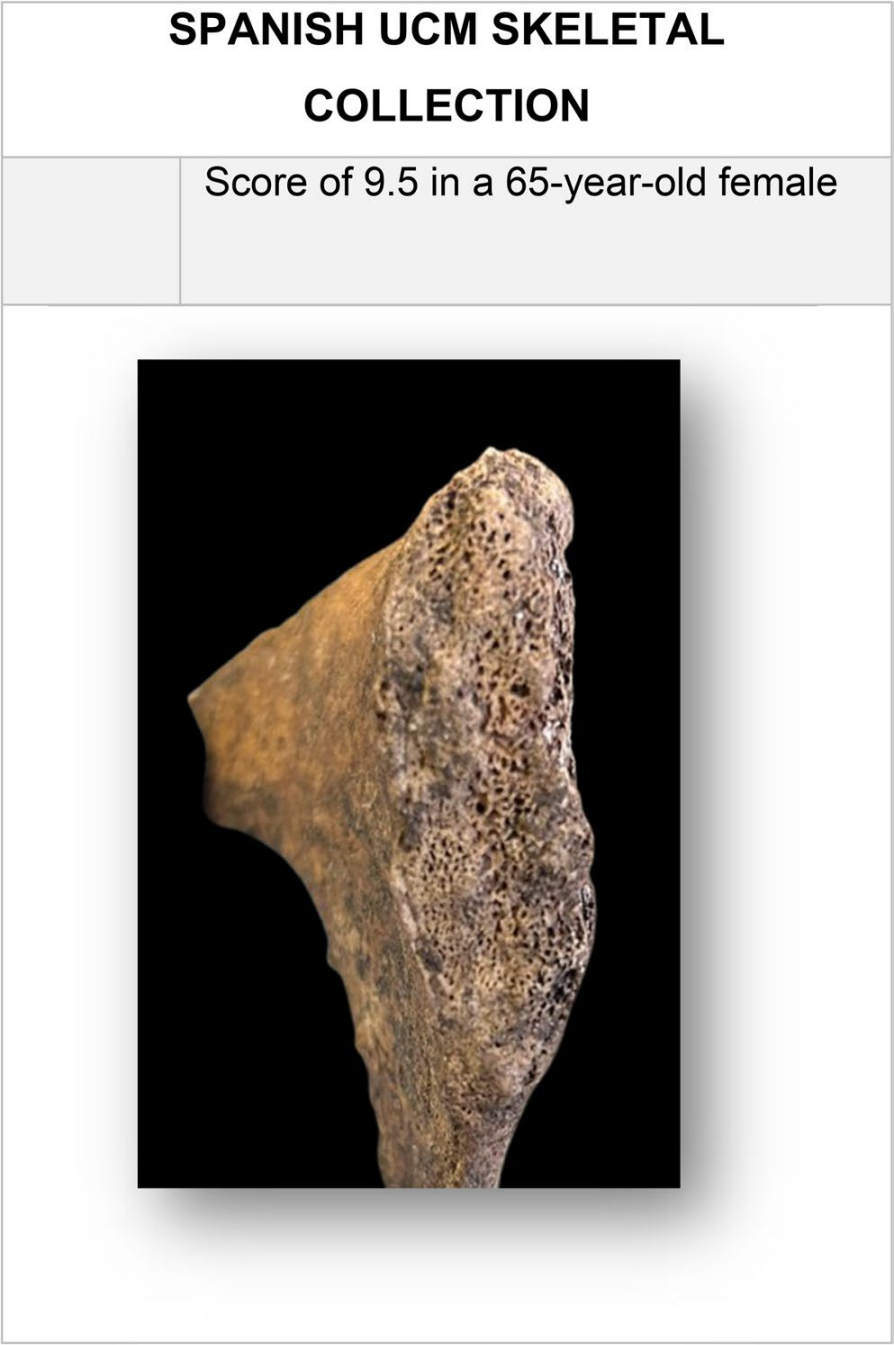
Score of 9.5 in a 65-year-old female

**Fig. 3:**
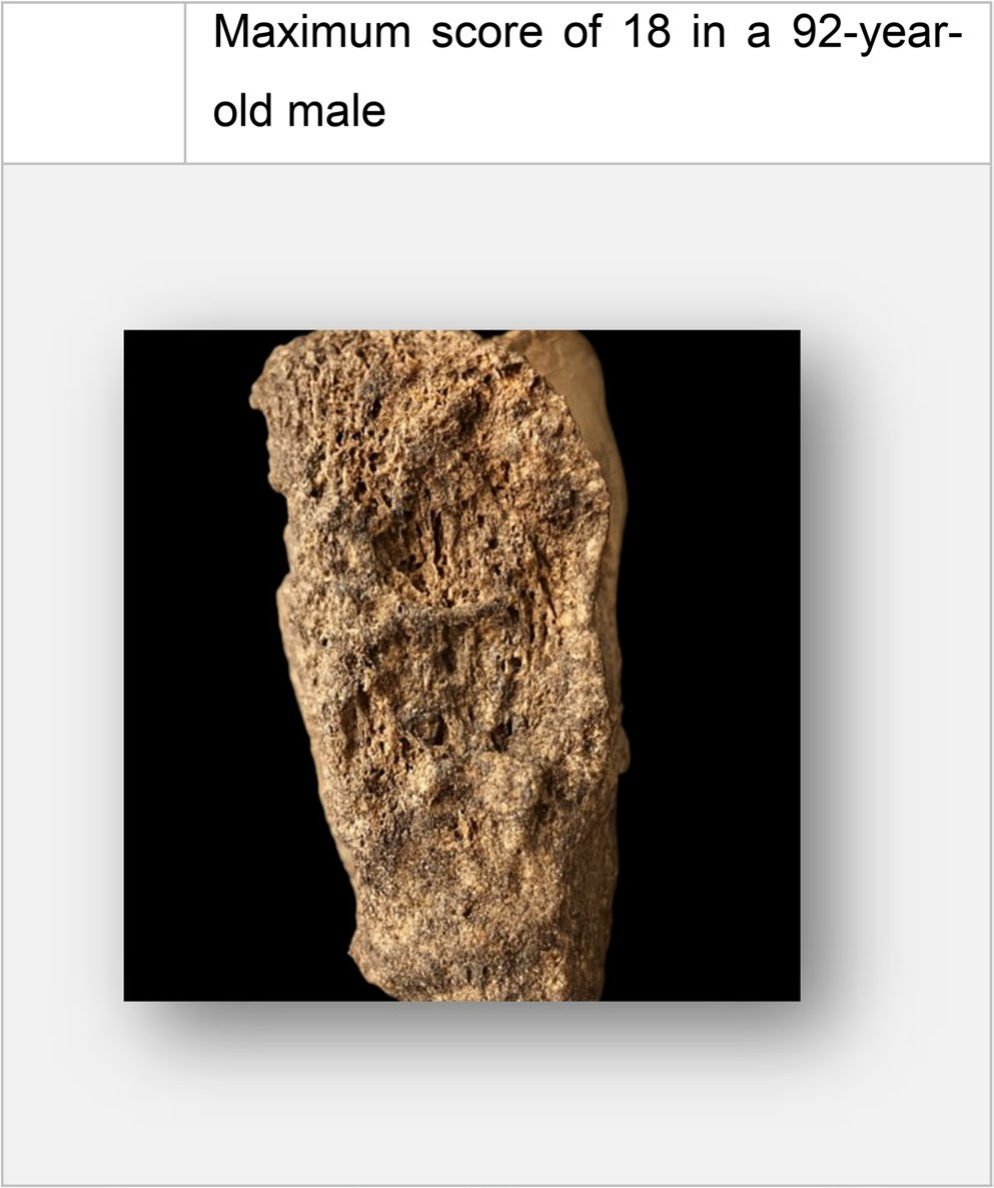
Maximum score of 18 in a 92-year-old male

**Fig. 4:**
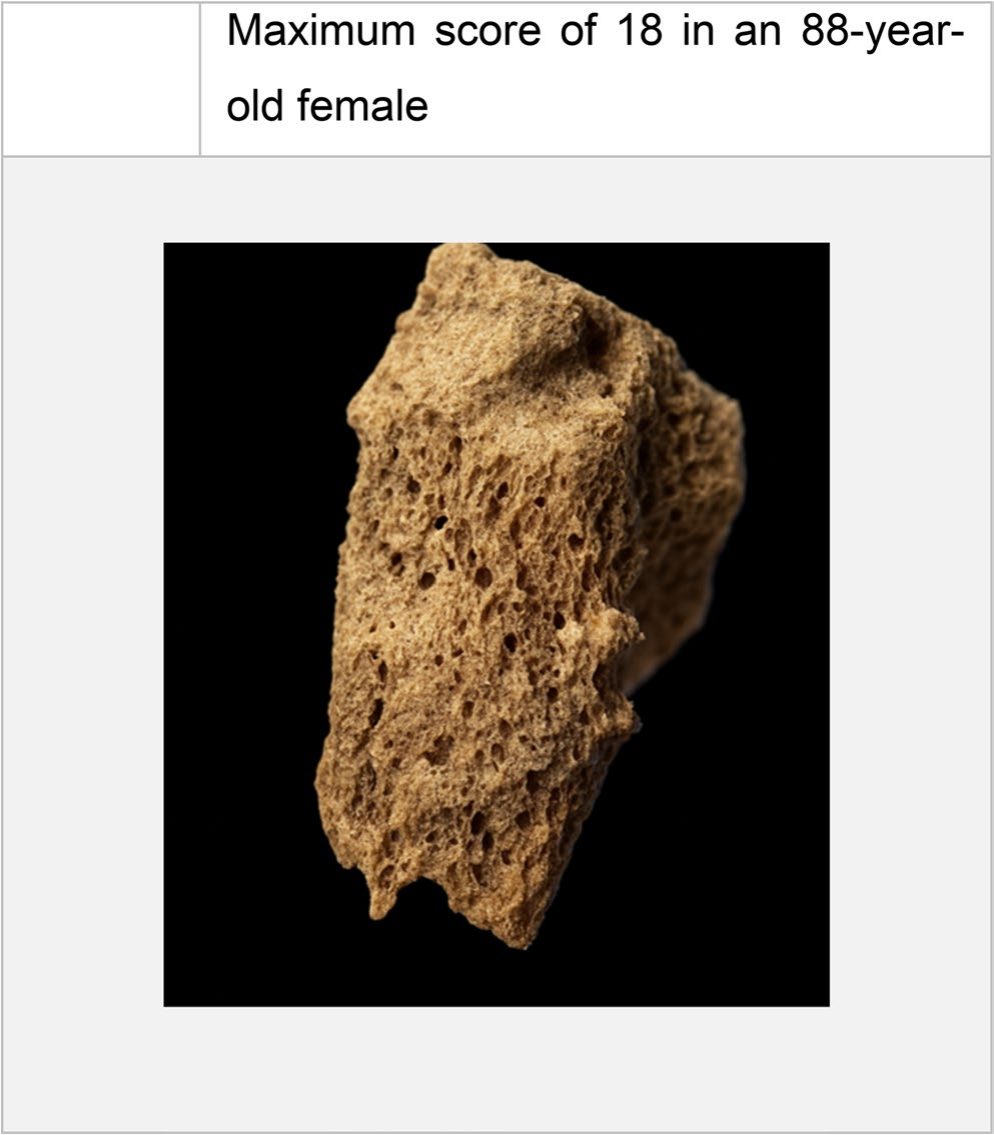
Maximum score of 18 in an 88-year-old female

Eight morphological traits of the pubic symphysis were evaluated, documented, and analyzed. The choice of features to be analyzed was based on those documented in the literature review [8, 11, 13, 16]. However, the present study extends previous work by evaluating not only the presence of each trait but also increasing severity or degeneration within each trait across older age groups. Each trait was assessed and scored using a structured point system developed by the author (Table 2). The composite total score ranges from 0 to 18. To capture transitional morphologies, half-scores (0.5) were permitted when a trait displayed partial expression (e.g., dorsal plateau partially flattened and partially deformed). A series of pubic symphysis images from both collections, UC and UCM, each with minimum and maximum score categorized by sex and age are presented below in Figs. 1, 2, 3 and 4.

**Table 2:** Summary of assessed traits and scoring system

Trait	Notes	Score
***Bone condition***	Refers to the overall quality and preservation.	0: Well-preserved 1: Moderate degradation 2: Advanced degradation with distortion 3: Severe degeneration and structural distortion.
***Surface porosity***	Indicates the presence and extent of pores or holes on the symphyseal face, with increased porosity typically linked to bone degeneration and aging.	0: None 1: Mild (scattered pores) 2: Moderate(widespread, larger pores) 3: Severe (deep, extensive porosity)
***Rim irregularity***	Describes the disruption of the outer edge of the symphyseal face. The rim can appear irregular, crenulated, eroded, chipped, or exhibit wizening with bone growth.	0: Regular margin 1: Mild irregularity 2: Severe lipping or erosion
***Ventral rampart integrity***	Refers to the condition of the ventral (anterior) border of the pubic symphysis, which may either remain intact or show signs of partial or total breakdown.	0: Intact 1: Partially lost 2: Complete resorption and bony excrescences
***Dorsal plateau shape***	Involves the form and contour of the dorsal surface; the presence of lipping can vary in severity.	0: Normal 1: Flattened 2: Collapsed/deformed
***Ectopic ossification***	Refers to the formation of bone in areas not normally ossified, particularly around the margins.	0: None 1: Small Nodules 2: Large osteophytes/bridging
***Surface texture***	Describes the degree of granularity, including the presence of micro/macro porosity or coarseness.	0: Smooth/flat 1: Granular or irregular 2: Heavily degraded, rugged
***Symphyseal face elevation***	Refers to whether the symphyseal surface is flat, depressed, or raised.	0: Flat 1: Slightly sunken or elevated 2: Severely distorted due to bony remodeling

#### Measurement of pelvic bone weight

Weight is an indicator that changes with age. The weight of individual bones is widely employed in various applications, including the analysis of archaeological cremations and forensic investigation. However, the relationship between bone mass and weight is highly variable and is influenced by several factors, invluding diet, mechanical loading, and muscle and genetic function [15]. In addition, bone weight may be influenced by preservation conditions, humidity, and taphonomic processes. To standardize the factors stated above and minimize potential bias, the samples were dry, curated skeletal collections housed in laboratory environments, thereby limiting variability related to moisture content and short-term environmental exposure. In general, the skeletal remains appeared to be in good condition of preservation, although some skeletal parts survived better than others. Remains exhibiting visible taphonomic alteration, mineral infiltration, or post-depositional damage were excluded from the analysis. However, long-term diagenetic effects cannot be fully eliminated in skeletal material, and this limitation is inherent to osteological research.

To evaluate whether pelvic bone weight correlates with advancing age, each innominate bone was weighed separately using a precision laboratory scale with a 0.1g margin of error. Left and right sides were recorded independently. A fully intact pelvis was excluded from weight assessment and omitted from the sample, as the complete pelvic bone made it difficult to visualize the symphyseal surface clearly. Accordingly, bone weight is interpreted here as a supporting quantitative variable, rather than a direct measure of bone density or an independent age estimator.

Weight was first measured in the Portuguese sample and then measured in the Spanish sample using identical procedures. These data were incorporated into regression analyses to determine whether weight contributed meaningfully to older age and late-adult phases.

All innominate bones were assessed independently by the primary author. After scoring was completed, the known ages were revealed, and individuals were grouped into clusters based on similarity in total scores, bone weights, and observed morphological patterns. These clusters formed the basis for revised late-adult phases analogous to, but more narrowly defined than, Suchey–Brooks Phase 6 (designated in this study as Phase 1) and the expanded Phase 7 (designated in this study as Phase 2) described by Berg and Hartnett.

## Statistical analyses

All statistical analyses were performed using R (Version 4.4.2; R Core Team, 2024) [32]. The analyses aimed to (1) refine the newly developed scoring system, (2) evaluate its performance within and between samples, and (3) assess its reliability and accuracy. The statistical procedures applied are described below.

### Analytical framework and phase determination

A regression tree analysis was first conducted on the Portuguese sample (UC) and then again on the Spanish sample (UCM) to identify natural groupings of individuals based on total degenerative score, pelvic bone weight, sex, and chronological age. The regression trees were constructed using the CART (Classification and Regression Trees) algorithm [33]. The authors applied a pre-pruning strategy by setting a complexity parameter to 0.01 and a decision stump architecture, restricting the tree to a maximum depth of 1 split (generating, for practical purposes, a single cutoff point). The authors chose this tree architecture because it reduces complexity, maximizing its applicability in different contexts. To ensure model robustness, the authors used a 70/30 train-test split across all datasets, effectively isolating the training process from the final evaluation (to prevent data leakage).

This analysis produced two distinct late-adult phases: Phase 1 and Phase 2, corresponding approximately to refined versions of Phases 6 and 7, respectively, of existing pubic symphysis methods. These phases were then applied to the Spanish sample (UCM). For each phase, descriptive statistics (mean, standard deviation, and age range) were generated separately for males and females in both samples. Histograms (1–4) illustrate the distribution of true ages within each phase, allowing visual assessment of phase distinctiveness and overlap.

### Comparison with existing models

To evaluate external validity, the resulting phase ranges of both samples were compared with published data from Suchey–Brooks [8], Hartnett (FSC and WBD samples) [16], and Berg [17]. This comparison (Table 7) assessed whether aging trajectories observed in Portuguese and Spanish elderly samples aligned with, or diverged from, existing reference populations, particularly in the upper phases.

### Bilateral asymmetry and prosthesis effects

To test for anatomical or scoring asymmetry, Wilcoxon signed-rank tests compared left vs. right pelvic bones (total score and weight) in both collections (UC and UCM), and prosthetic vs. non-prosthetic sides within the Portuguese sample. These analyses determined whether unilateral hip prostheses or natural asymmetry influenced degenerative scores or bone weight. The Spanish sample did not contain prosthesis cases and was therefore excluded from this specific analysis.

### Bone weight, age, and sex differences

Associations between pelvic bone weight and chronological age were evaluated using Pearson correlation coefficients calculated separately for males, females, and the combined sample, in both Portuguese and Spanish datasets. Boxplots (Plots 1–6) depict the observed negative correlations, as well as sex-based differences in bone weight distribution. These analyses provided the quantitative foundation for integrating bone weight into late-adult phase refinement.

### Inter- and intra-observer reliability

Method reproducibility was assessed using Spearman’s rank correlation coefficients and Intraclass Correlation Coefficients. A total of 15 pelvic bones per collection (UC and UCM) were independently scored by the primary author (two scoring rounds, separated by three weeks). Four additional observers (two observers from the Forensic Anthropology Laboratory, Coimbra, Portugal- one with more than 20 years of experience in forensic anthropology and one doctoral student in forensic anthropology, and two observers from the Forensic Anthropology Laboratory at the Department of Legal Medicine, UCM, both with more than 20 years of professional experience.

Intra-observer consistency (primary author) and inter-observer agreement (primary author vs. other observers) were calculated to evaluate robustness and replicability of the scoring procedure.

### Accuracy assessment: bias and inaccuracy

To quantify overall methodological performance, bias and inaccuracy were calculated using standard formulas:

- Inaccuracy = ∑∣estimated age−known age∣n\frac{\sum |\text{estimated age} - \text{known age}|}{n}n∑∣estimated age−known age∣.
- Bias = ∑(estimated age−known age)n\frac{\sum (\text{estimated age} - \text{known age})}{n}n∑(estimated age−known age).

In both formulas, “estimated age” refers to the median age for the phase to which individuals belong. These values were computed for Phase 1 and 2, seperately for males and females, and for both Portuguese and Spanish samples. For the Portuguese collection, calculations used a random 30% subsample; for the Spanish sample, the full dataset was analyzed. Results are presented in Tables 7 and 8. Confidence Intervals (95%) were also calculated for both bias and inaccuracy.

## Results

### Descriptive & regression statistics

Regression tree analysis identified two distinct late-adult phases in both the Portuguese (UC) and Spanish (UCM) samples, with patterns evaluated separately for males and females. These phases broadly correspond to refined versions of Suchey & Hartnett’s Phase 6 (Phase 1) and Hartnett’s Phase 7 (Phase 2) in traditional pubic symphysis aging systems.

In the Portuguese male sample, Phase 1(purple) included individuals aged 60–87.5 years, while those in Phase 2(yellow) comprised individuals aged 70 years to 94+ (Histogram 1, Table 3). Among Portuguese females, Phase 1 encompassed ages 60–97 years, and Phase 2 included 75–97 + years, reflecting broader morphological variability (Table 4, Histogram 2).

**Table 3:** Descriptive Statistics, Phase Groupings, and Score Characteristics for Portuguese and Spanish **Male** Samples

	Phase	n	Mean	SD	Range	Total Score	Weight (in grams)	Split point
UCM	**1**	14	72.5	7.60	60–85	6.5–13	87.25–188.65	15.5
	**2**	17	85	5.48	76–94	14–18	84.2–187.8	
UC	**1**	29	73.75	19.44	60–87.5.5	4–10	101.50–265.08	13.5
	**2**	30	82	16.97	70–94+	11–18	68.64–207.15	

UCM, Universidad Complutense De Madrid

UC, Universidade De Coimbra

90% Confidence Interval

**Table 4:** Descriptive Statistics, Phase Groupings, and Score Characteristics for Portuguese and Spanish **Female** Samples

	Phase	n	Mean	SD	Range	Total Score	Weight (in grams)	Split point
UCM	**1**	25	71.5	3.45	66–83	6–13	60–121.75	13.5
	**2**	18	81	6.63	70–92	14–18	37–113	
UC	**1**	57	78.5	26.16	60–97	2.05–10	61.75–190.65	10.5
	**2**	20	86	15.56	75–97	11–18	60–158	

UCM, Universidad Complutense De Madrid

UC, Universidade De Coimbra

90% Confidence Interval

**Histogram 1:**
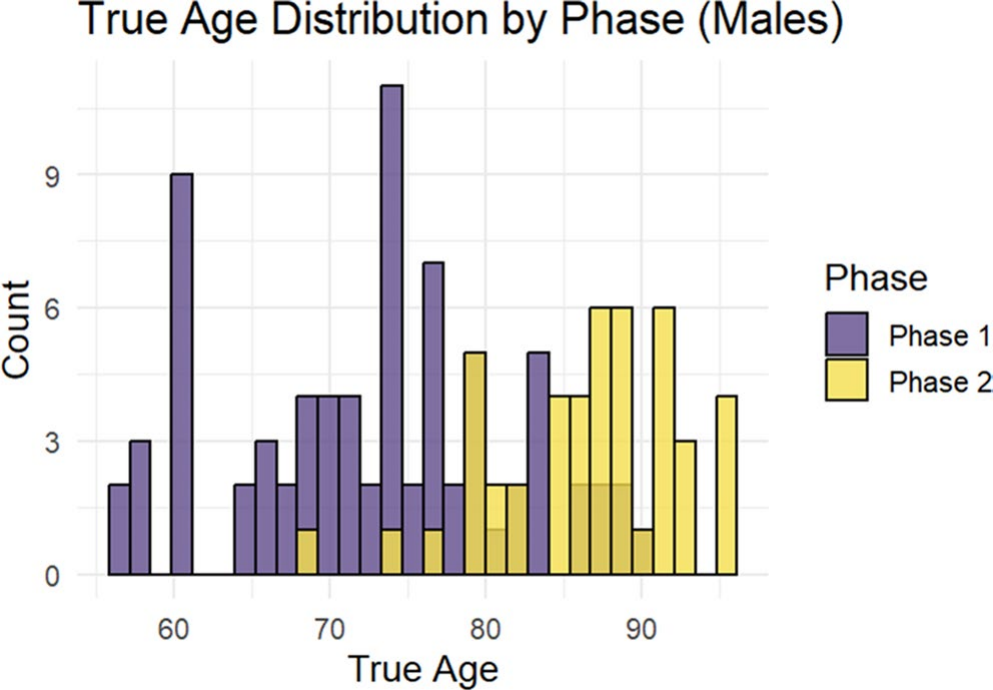
True age distribution by phase (P. Males)

**Histogram 2:**
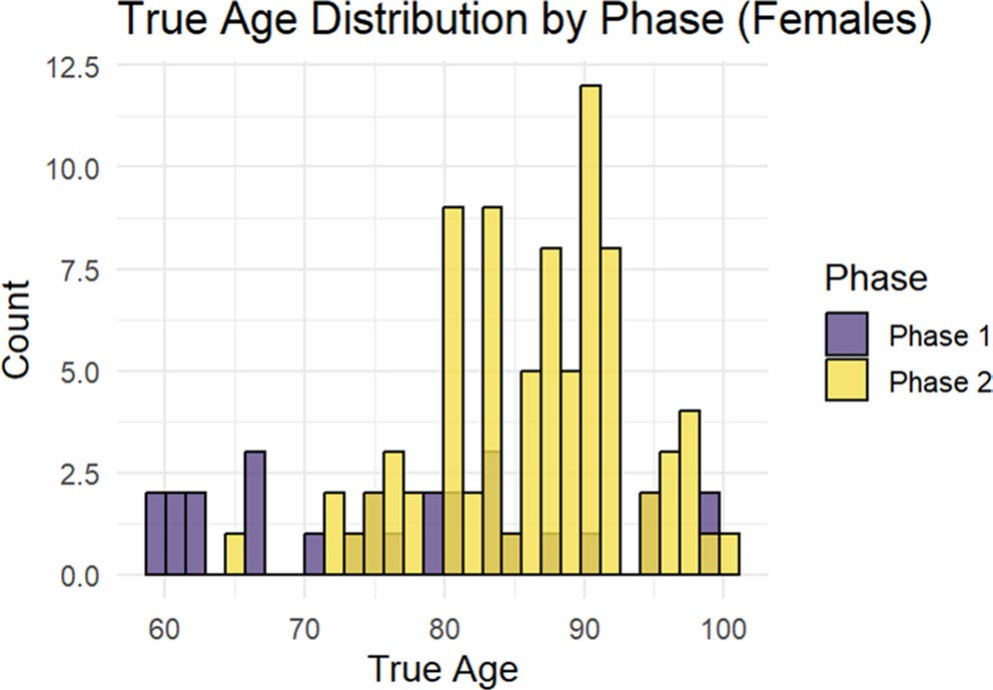
True age distribution by phase (P. Females)

**Histogram 3:**
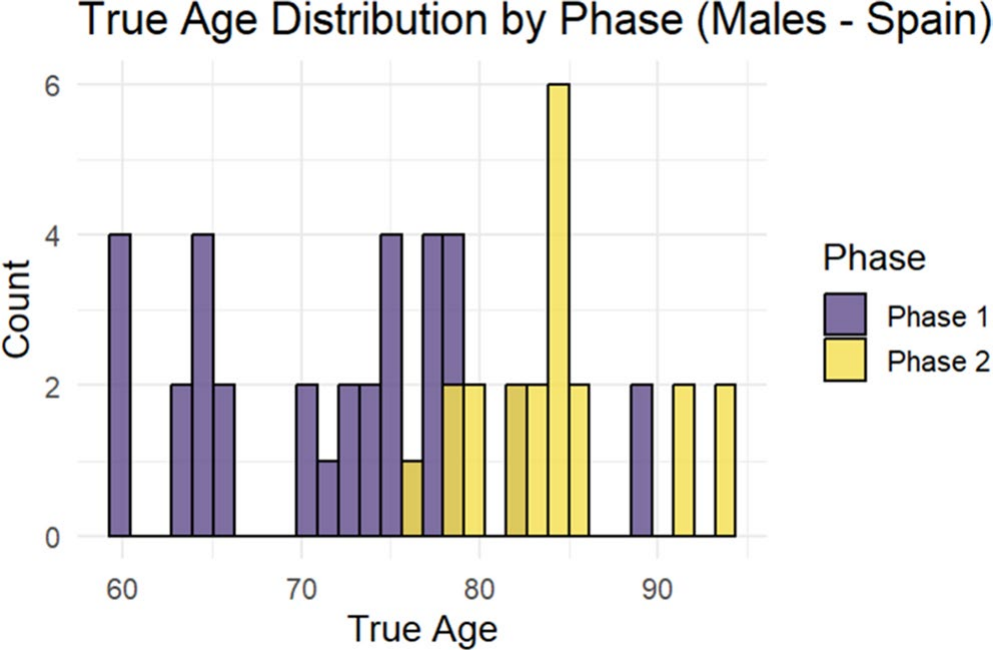
True age distribution by phase (S. Males)

**Histogram 4:**
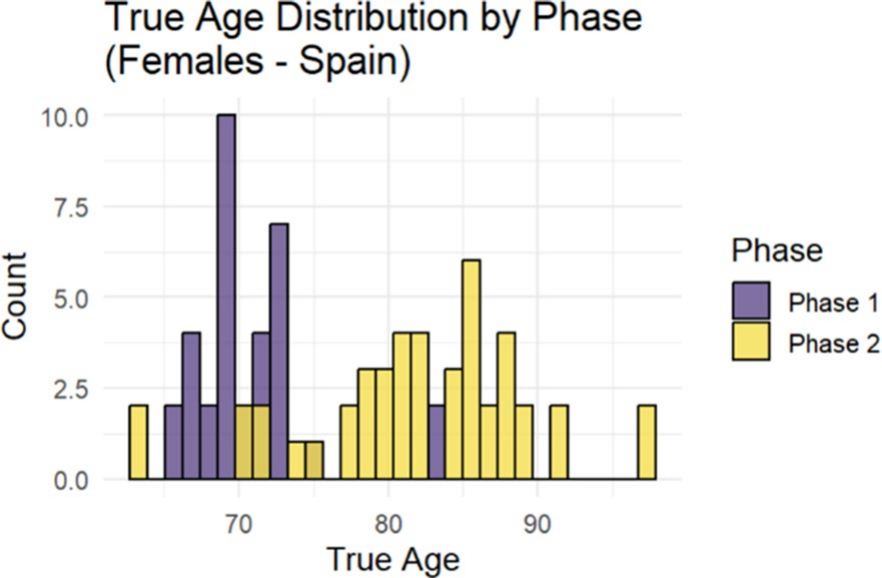
True age distribution by phase (S. Females)

The Spanish male sample exhibited Phase 1(purple) ages of 60–85 years, and Phase 2(yellow) ages of 76–94 years (Table 5, Histogram 3). Spanish females in Phase 1 ranged from 66 to 77 years, while Phase 2 ranged from 70 to 92 + years (Table 3, Histogram 4). Collectively, these results demonstrate consistent phase separation across both samples from both collections (UC and UCM), albeit with slightly narrower ranges in the Spanish dataset due to a smaller sample size.

**Table 5:** Age Phases: Comparative overview of UC, UCM, Suchey-Brooks, Hartnett, and Berg

	Phase	Mean Age (years)	Age Range (years)
UC COLLECTION	Group 1/Phase 6	73.75	60–87.5 (Males)
*UC COLLECTION*	Group 2/Phase 7	82	70–94+ (Males)
*UC COLLECTION*	Group 1/Phase 6	78.5	60–97 (Females)
*UC COLLECTION*	Group 2/Phase 7	86	75–97 (Females)
*UCM COLLECTION*	Group 1/Phase 6	72.5	60–85 (Males)
*UCM COLLECTION*	Group 2/Phase 7	85	76–94 (Males)
*UCM COLLECTION*	Group 1/Phase 6	71.5	66–83 (Females)
*UCM COLLECTION*	Group 2/Phase 7	81	70–92 (Females)
*SUCHEY- BROOKS 1990*	Phase 6	60.0 61.2	42–87 (Females) 34–86 (Males)
*SUCHEY-BROOKS 1990*	Phase 7	-	-
Hartnett 2010- *FSC* Hartnett 2010- *WBD*	Phase 6	72.34 64.2	56–86 (males and females) -
Hartnett 2010- *FSC* Hartnett 2010- *WBD*	Phase 7	82.54 74.2	62–99 (males and females) -
Berg 2008	Phase 6	56	-
Berg 2008	Phase 7	74.4	-

#### Comparison with prior aging systems

To contextualize the Portuguese and Spanish phases by sex, results were compared with published reference data from Suchey–Brooks, Hartnett (FSC and WBD samples) [16], and Berg [17] (Table 5). For males, Hartnett’s FSC sample mean ages phase 6 and 7, respectively, (around 72 and 82 years) closely match those of the Portuguese UC (73.75 and 82 years) and Spanish UCM (72.5 and 85 years) samples, but are higher than Hartnett’s WBD sample (64.2 and 74.2 years). For females, the Portuguese UC sample shows slightly higher mean ages (78.5 and 86 years) than the Spanish UCM (71.5 and 81 years) and Hartnett’s FSC (72.34 and 82.54 years), but exceeding WBD (64.2 and 74.2 years) female samples. Finally, Berg’s phases 6 and 7 show notably lower mean ages (56 and 74.4 years), particularly for Phase 6, compared to all samples used in this study. In both samples, Phase 1 exhibited higher bone weights than Phase 2, consistent with progressive trabecular and cortical bone loss in advanced age. This pattern supports integrating of bone weight as a supplementary parameter in late-adult phase assignment.

#### Bilateral asymmetry & prosthesis effects

Bilateral comparisons using the Wilcoxon signed-rank test revealed no significant differences between left and right sides for either total degenerative score or pelvic bone weight in both the Portuguese and Spanish samples (UC weight *p* = 0.086; UC score *p* = 0.793; UCM score *p* = 0.33; UCM weight *p* = 0.71). This confirms that degenerative scoring is not side-dependent. Among Portuguese individuals with hip prostheses, comparisons between prosthetic and non-prosthetic sides likewise showed no significant differences in bone weight (*p* = 1.000) or total score (*p* = 0.581). Additional tests demonstrated no meaningful associations between prosthesis presence and bone weight (*p* = 0.054) or between prosthesis score and total score (*p* = 0.796). These results indicate that prosthetic implants did not introduce measurable asymmetry or bias. 

#### Bone weight, age correlations, and sex differences

Pearson’s Correlation showed a negative correlation between age and pelvic bone weight across the Portuguese sample: females had a correlation coefficient (cor) of − 0.426, males − 0.33, and the total sample − 0.44, indicating that pelvic bone weight decreases with increasing age as seen in Plot 1 (female) and Plot 2 (male). Male pelvic bones were consistently heavier than those of females, with mean weights of approximately 150 g for males and 100 g for females (Plot 3). Applying the same analysis to the Spanish sample produced comparable results: correlation values of − 0.363 for females, − 0.331 for males, and − 0.27 for the total sample. Again, pelvic bone weight decreased with age, as shown in Plot 4 (females) and Plot 5 (males). As with the Portuguese sample, males displayed higher bone weights than females, with average values of roughly 130 g for males and 80 g for females (Plot 6).

**Plot 1:**
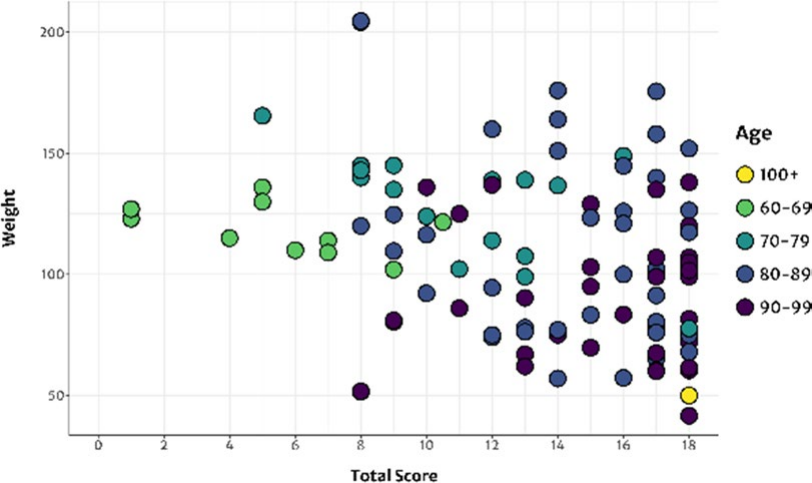
Pelvic bone weight in relation to age – Females – Portuguese sample

**Plot 2:**
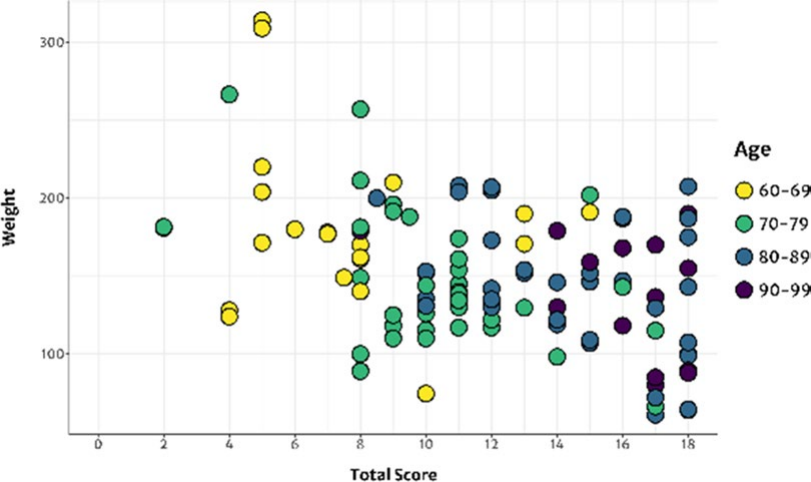
Pelvic bone weight in relation to age – Males – Portuguese sample

**Plot 3:**
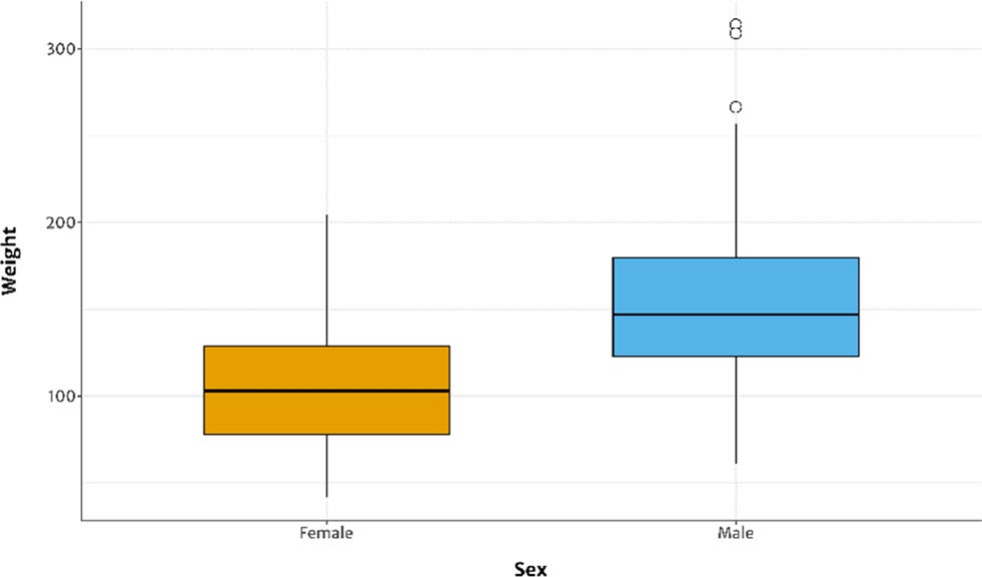
Boxplot of Pelvic Bone Weight Stratified by Sex – Portuguese sample

**Plot 4:**
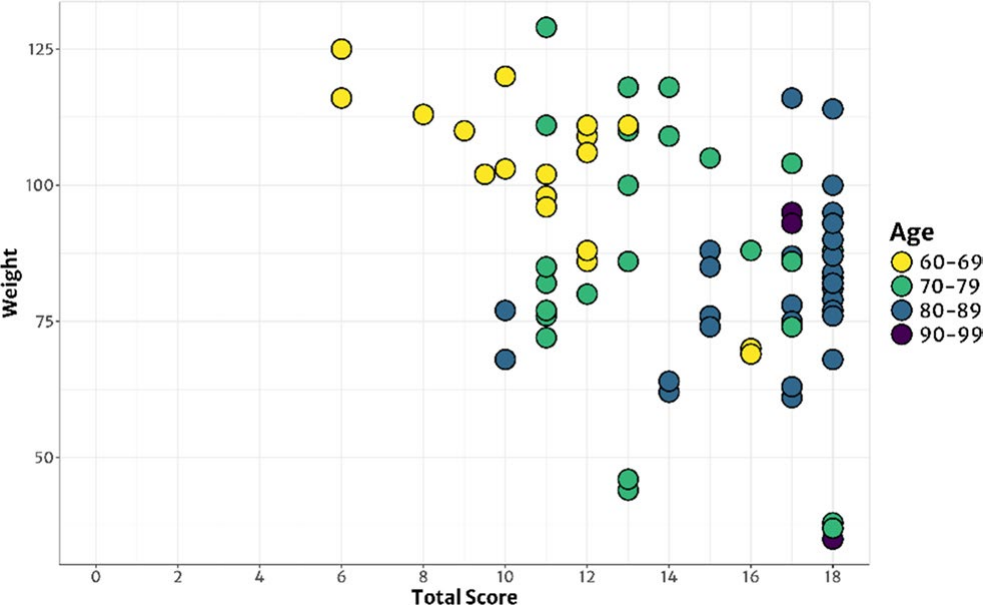
Pelvic bone weight in relation to age – Females – Spanish sample

**Plot 5:**
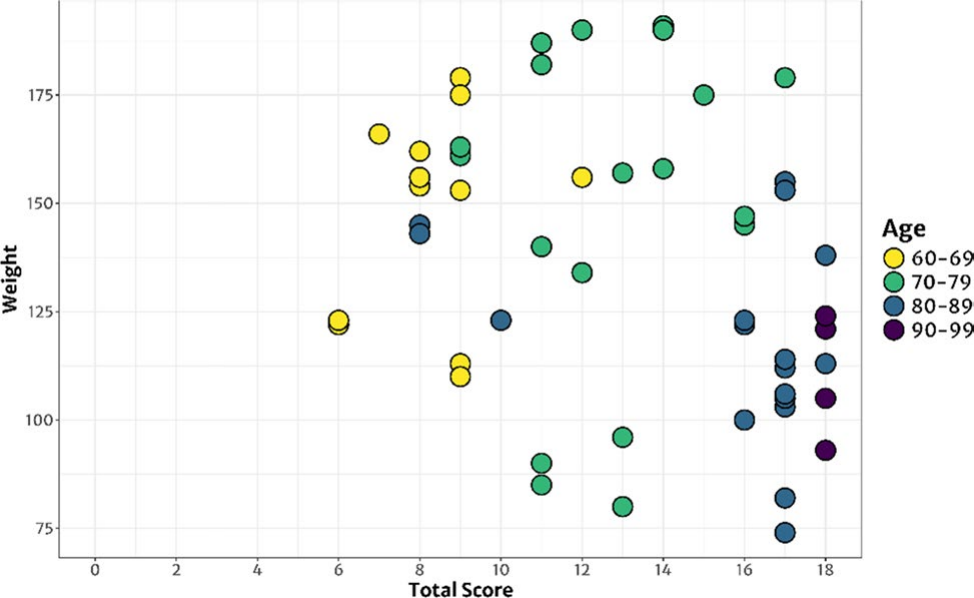
Pelvic bone weight in relation to age – Males – Spanish sample

**Plot 6:**
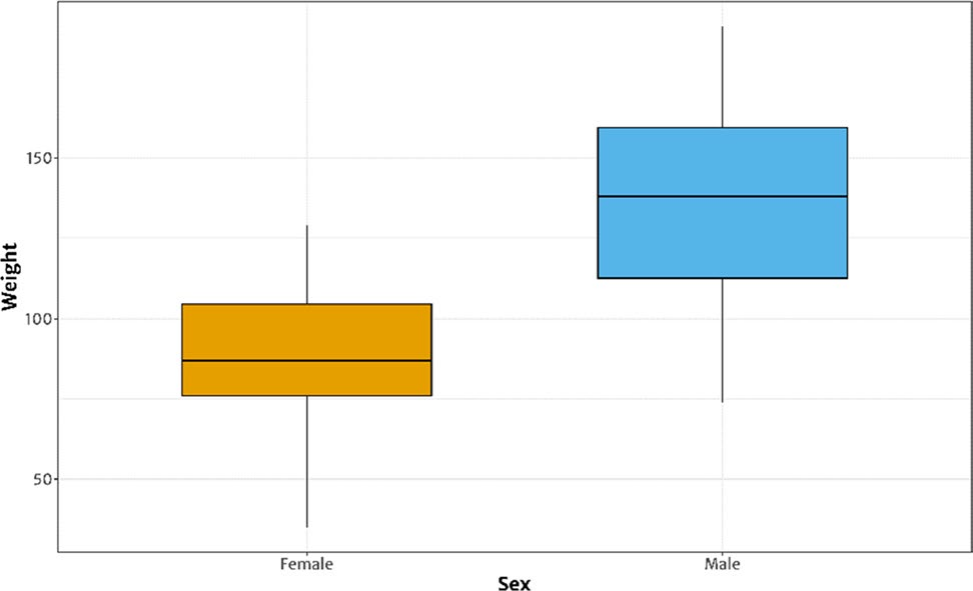
Boxplot of Pelvic Bone Weight Stratified by Sex – Spanish sample

#### Inter & intra observer test

The intra-observer analysis, presented in Table 6, showed Spearman’s rho values of 0.77 for the Portuguese sample and 0.97 for the Spanish sample, reflecting strong consistency in repeated age assessments conducted by the primary author. Inter-observer comparisons (see Table 6) with the primary author demonstrated moderate to strong agreement across both observers, with Observer 2 showing the highest correlation for the Portuguese sample (ρ = 0.75). For the Spanish sample, inter-observer reliability similarly indicated strong agreement, with the highest correlation observed between Observer 1 and the primary author (ρ = 0.91). Variability in agreement levels across observers may be attributable to differences in experience and training.

**Table 6:** Spearman’s Rank Correlation Coefficients and Intraclass Correlation Coefficient, indicating Intra- and Inter-Observer Reliability Among Observers – Portuguese & Spanish collection

Observers	Portuguese collection	Spanish collection
	Rho	rho
Cindy1 / Cindy2	0.77	0.98
Cindy2 / Observer1	0.67	0.91
Cindy2 / Observer2	0.75	0.77
Observer1/Observer2	0.59	0.90

#### Bias and inaccuracy

Bias and inaccuracy in the UC and UCM collection processes across two phases, stratified by sex, are presented in Tables 7 and 8, respectively. Phase 1 of the UC collection, bias was negative for both males (− 2.21) and females (− 4.75), with females showing a larger magnitude of bias. Inaccuracy was higher in females (8.12) than in males (6.79). In Phase 2 of the UC collection, male bias remained negative (− 3.33), whereas female bias shifted to positive (3.12). Inaccuracy decreased for both sexes compared to Phase 1, with values of 5.17 for males and 6.80 for females.

**Table 7:** Bias and Inaccuracy from the UC Collection by sex and phase

Phase	Bias		Inaccuracy	
	Male	Female	Male	Female
Phase 1	−2.21	−4.75	6.79	8.12
Phase 2	−3.33	3.12	5.17	6.8

**Table 8:** Bias and Inaccuracy from the UCM Collection by sex and phase

Phase	Bias		Inaccuracy	
	Male	Female	Male	Female
Phase 1	1.59	0.889	4.95	1.78
Phase 2	2.86	2.6	3.43	5.67

## Discussion

This study aimed to explore advanced pubic symphyseal features, develop a standardized quantitative scoring system for their assessment, and examine pelvic bone weight as a variable associated with age and degenerative change. It further refined the upper-age phases of the Suchey–Brooks [8], Hartnett [16], and Berg [17] systems by implementing more specific diagnostic criteria and narrower age ranges. Although these methods allow for successful discrimination between individuals over age 60 years and under age 60 years, they may still be limited, given the increase in life expectancy [15]. Commonly described characteristics of the pubic symphysis in advanced age include marked ventral ligamentous outgrowths, pitting or porosity of the articular face, crenulation, and irregularity or depression of the symphyseal surface as the rim undergoes erosion. However, individuals aged 60 - 100 years do not exhibit a uniform pattern of degeneration. The degree and expression of these traits are not identical for someone aged 60, 70, 80, 90, or 100 years. This study demonstrates that individuals aged 60 and older can be classified, including the estimation of those with a high likelihood of being 70 or 80 years of age or older. While taphonomic processes may modify the surface postmortem, the intrinsic biological aging, particularly bone loss and structural remodeling, drives predictable morphological changes that evolve over time . In cases involving the recovery of human remains, the pelvic region has been shown to exhibit the highest survival rate, regardless of the depositional context, as reported by Capella et al. in 2017 [34]. However, its condition may be affected by scavenger activity in outdoor recovery contexts, damage caused by soil processes or other taphonomic factors in buried remains, and exposure to fire in cases involving burning. While the pubic symphysis is typically well preserved in buried skeletons, it is frequently subject to gnawing by predators in outdoor environments. This study assessed taphonomy, and taphonomically good samples were selected for this study.

This study reflects transitional states, as did Buckberry and Chamberlain [35] for the auricular surface, and Truesdell for the pubic symphysis [11]. In addition, it incorporates pelvic bone weight as a quantitative variable in age estimation for older adults. Although research on bone weight dates to the 19th century [36] and has been employed to examine population and sex differences as well as pathological effects [37–39], only Hartnett has recently applied it to the pubic symphysis, reporting that pubic weight decreases with age. By contrast, Teixeira and Cunha [40] studied on cranial traits and found no significance between cranial weight and advanced age. Introducing a quantitative measure of pelvic bone weight in this study offers significant advantages over the qualitative descriptors (e.g., “heavy” or “light”) employed by Hartnett [16], which helps the investigator move between phases 6 and 7. Numerical weight values provide an objective, reproducible metric that minimizes observer bias and improves consistency across practitioners. Unlike qualitative categories, which are inherently subjective and coarse, quantitative measurements capture subtle variation in bone mass and can be incorporated into statistical or multivariate models. The present study evaluates the association between pelvic bone weight, chronological age, and morphological degeneration. Our results show that pelvic bone weight generally decreases with increasing degeneration and advancing age, though it should not be used as a standalone indicator. Instead, it provides an additional dimension for forensic anthropologists to understand sex- and age-related differences.

### Patterns observed in the portuguese sample

The Portuguese data revealed clear correlations: increasing degenerative scores and decreasing pelvic bone weight were broadly associated with advancing age. Sex-based weight differences were pronounced, and they align with weight patterns observed by Silva, Crubézy, and Cunha [15], where males over 60 weighed between 0.081 and 0.307 g and females between 0.033 and 0.197 g, showing that males weigh more than females. The earlier onset of osteoporosis in women, along with factors such as menopause, hormone fluctuations, parity, and loss of estrogen’s protective effects on bone mass, likely explains the overlap between Phase 1 and Phase 2 female weights. Since trabecular bone is more susceptible to rapid turnover than cortical bone, postmenopausal skeletal changes can create appearances suggestive of advanced age even in relatively younger women. Men, by contrast, experience slower age-related bone loss, losing roughly two-thirds of the cortical and trabecular bone lost by women over a lifetime [41]. Although bone weight is affected by age-related reductions in bone mass, where relatively low bone weight often reflects increased osteopenia, as noted by Berg [17], it can also be modified by preservation conditions, postmortem alterations, and taphonomic processes, as cautioned by Hartnett [16].

### Findings from the spanish sample

**Fig. 5:**
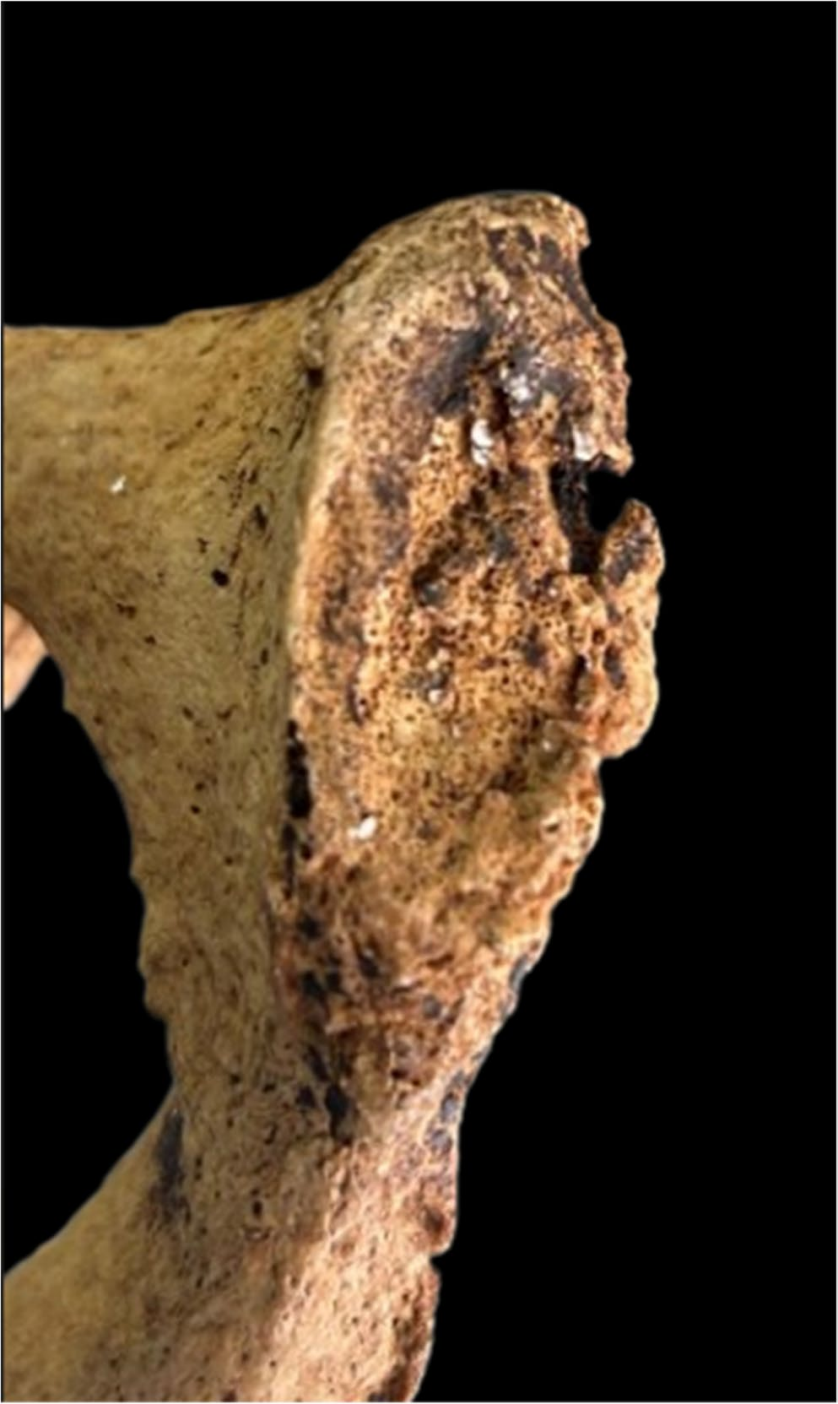
Score of 15 in a 73-year-old male

**Fig. 6:**
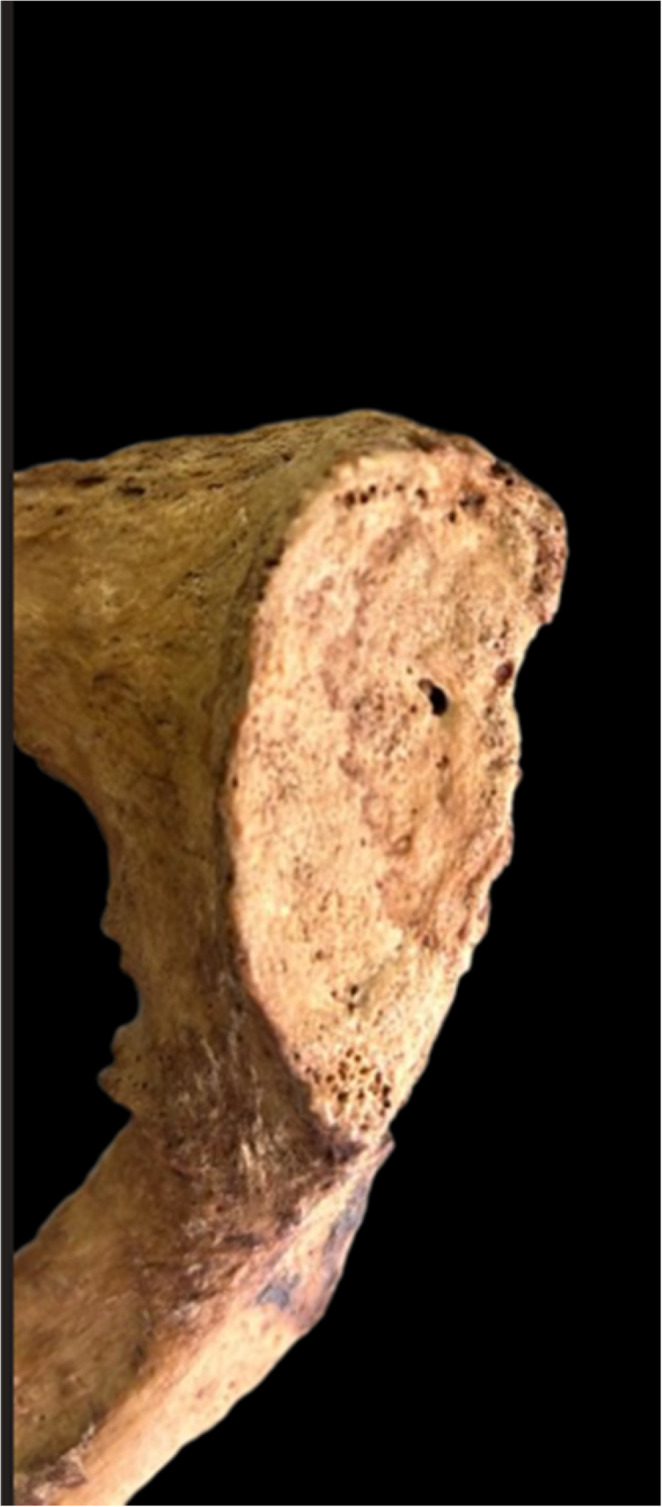
Score of 8 in an 89-year-old male

Spanish males’ phases displayed trends similar to the Portuguese sample; however, Spanish females’ phases showed an atypical inversion. This suggests that the regression-tree separation algorithm may have been more sensitive to morphological change than to chronological age in this subgroup. Factors such as accelerated skeletal aging, sample size, and hormonal differences likely contributed to this deviation. Case examples illustrate this variability: an 89-year-old male scored only 8 points (Fig. 5), reflecting relatively youthful morphology, whereas a 75-year-old male scored 15 points (Fig. 6), indicating accelerated degeneration. These contrasts emphasize that individual variation, environment, genetics, and lifestyle can influence the pace of skeletal aging.

The lack of a clear age–weight gradient in Spanish females resembles patterns described by Cho et al. in 2006 [42], who reported population-specific trajectories in bone mass maintenance; European American males tended to preserve bone mass with age, whereas other groups, including African-American males, exhibited age-related bone loss. However, the Portuguese sample demonstrated a consistent relationship between higher degenerative scores, lower bone weight, and increasing age, mirroring trends observed by Eugenia and colleagues. Bone weight, therefore, provides an additional layer of analytical resolution. As Berg [17] noted, weight assessment is most helpful in borderline cases, particularly when the proportion of compact to porous bone approaches equivalence. Supporting this, Bascou [43] found a significant negative correlation between bone density and age in CT-based analyses, and Wink [44] emphasized the diagnostic value of bone weight, texture, and tactile qualities in older adults. The moderate negative correlation observed in this study (*r* = − 0.44 overall; − 0.426 in females; − 0.33 in males) confirms that pelvic weight serves as a useful supplementary variable, particularly given that previous research rarely quantified this relationship.

Importantly, the method developed in this study demonstrated strong reliability and high reproducibility, given the Spearman’s rho values of 0.77 for the Portuguese sample and 0.98 for the Spanish sample, but still consistent with previously published standards, such as Hartnett’s reported value of 0.89 and Merritt’s interobserver agreement of κ = 0.849.

Interobserver agreement within the Portuguese sample (ρ = 0.67 and 0.75) and the Spanish sample (ρ = 0.91 and 0.77) falls within or slightly exceeds the range reported for comparable morphological aging methods. For instance, Berg [17] documented interobserver Spearman’s rank correlation coefficients typically between 0.72 and 0.89, with values approaching 0.89 for highly experienced observers and ranging from approximately 0.72 to 0.80 when less experienced evaluators were included, which explains here the limitation of a less experienced observer, evaluating the Portuguese sample.

Bias and inaccuracy measures in this study further validate the approach. In the Portuguese sample (Table 7), Phase 1 females exhibited higher bias (− 4.75) and inaccuracy (8.12) than males (− 2.21 bias; 6.79 inaccuracy), possibly reflecting greater biological variability or skeletal fragility. In Phase 2, male bias remained negative (− 3.33), while female bias shifted to positive (+ 3.12), suggesting that older women were more likely to be overaged. In contrast, Spanish females had more consistent results: lower bias (0.88) and inaccuracy (1.78) in Phase 1, though in Phase 2, inaccuracy increased to 5.67. This pattern suggests that the method may be particularly suited to younger elderly females but requires caution when applied to older females due to higher biological variability.

In the Portuguese sample, greater error among females in Phase 1 may reflect increased biological variability or skeletal fragility, which can complicate age estimation. The shift observed in Phase 2-from underestimation in males to overestimation in females- suggests a systematic tendency to overage older women, potentially due to age-related skeletal changes progressing differently by sex. In contrast, Spanish females showed more stable performance in Phase 1, indicating that the method performs particularly well for younger elderly females in this population. However, the rise in inaccuracy in Phase 2 underscores the need for caution when applying the method to older age groups, where cumulative biological variability may reduce precision. This has been stated by Priya in 2017 [45], which notes that in pubic symphysis aging methods, morphological variability is greater in females, e.g., due to parity effects, which complicates the direct application of male-based standards to female samples. The presence of a femoral prosthesis does not substantially influence pubic symphyseal morphology nor its weight (Portuguese sample), and this aligns with earlier results reported by Janamarie Truesdell [36], indicating only minimal differences between skeletal scores of individuals with and without prostheses. Body weight showed marginal significance (*p* = 0.054), indicating a potential but inconclusive trend. These observations underscore the need for further research employing larger or more stratified samples, especially given the scarcity of studies examining the effects of prosthetic devices on age-estimation methods.

### Limitations

From a forensic standpoint, the development of a quantitative scoring system and refined upper-age criteria enhances the precision of late-adult age-at-death estimation. This is particularly valuable in casework, where overly broad upper-age categories may hinder identification efforts. These results should be interpreted with consideration of population specificity, sample composition, and the potential impact of taphonomic alteration on surface morphology. The findings must be interpreted with particular caution due to the relatively small sample size (*n* = 228 individuals > 60 years of age from both collections UC and UCM), which is especially consequential given the study’s implications for forensic casework and age-at-death estimation in older adults. This restricted sample size may limit the applicability of the results and the robustness of statistical inferences, particularly for advanced age groups. Despite these limitations, the study is a good starting point in the study of skeletal age estimation of older individuals in accordance with combining both morphological and quantitative techniques. The study also provides valuable preliminary insights into age-related morphological changes in elderly individuals and establishes a foundation for future research using larger, more diverse skeletal samples to test and refine the proposed methods.

## Conclusion

This study presents a refined approach to age estimation in elderly adults by integrating a standardized morphological scoring system with pelvic bone weight, calibrated using two contemporary skeletal collections from Spain and Portugal. The method improves upon existing phase-based systems by providing narrower, diagnostic criteria for advanced adult age, particularly within the upper phases traditionally associated with substantial variability and reduced accuracy.

The incorporation of eight morphological traits, supplemented by quantitative bone weight, enhances the capacity to distinguish between late-adult phases and mitigates the well-documented underestimation of elderly individuals in widely used systems such as Suchey–Brooks. The refined phases developed here, analogous to updated versions of Phases 6 and 7, demonstrate strong applicability to both Portuguese and Spanish samples, with moderate to high reliability and acceptable levels of bias and inaccuracy. Importantly, the method performs consistently across sexes, and it is not affected by the presence of unilateral hip prostheses. By addressing a critical gap in late-adult age estimation and supporting the need for population-specific standards, this study contributes a robust, reproducible framework for forensic practitioners working with elderly individuals. Further validation of the proposed method on a second reference collection strengthens the study. Additionally, larger samples, 3D morphometric, histological or biochemical markers, and the integration of other anatomical sites and methods could be essential to advance the precision and generalizability of methods in advanced ages.

## Data Availability

The data presented in this study are available on reasonable request from the corresponding author.
